# Minimally Invasive Endoscopic Transorbital Approach for Frontal Sinus Fractures: A Comparative Study

**DOI:** 10.3390/cmtr18030041

**Published:** 2025-09-22

**Authors:** Laurence Verstraete, Paulien Schillemans, Jan Meeus, Philippe Vuylsteke, Robin Willaert

**Affiliations:** 1 Department of Oral and Maxillofacial Surgery, University Hospitals Leuven, Kapucijnenvoer 33, BE-3000 Leuven, Belgium; paulien.schillemans@uzleuven.be (P.S.); jan.meeus@uzleuven.be (J.M.); robin.willaert@uzleuven.be (R.W.); 2 Department of Oral and Maxillofacial Surgery, AZ Delta, Deltalaan 1, BE-8800 Roeselare, Belgium; philippe.vuylsteke@azdelta.be; 3 OMFS IMPATH Research Group, Department of Imaging and Pathology, Faculty of Medicine, KU Leuven, Kapucijnenvoer 33, BE-3000 Leuven, Belgium

**Keywords:** frontal sinus fracture, transorbital approach, minimally invasive, open fracture reduction

## Abstract

Background: This study aims to evaluate the use of the endoscopic transorbital approach for reducing frontal sinus fractures and compare its outcomes with the traditional bicoronal approach. Methods: A retrospective comparative analysis of case studies including all patients with frontal sinus fractures treated at our institution between January 2013 and December 2023 was conducted. Patients were categorized based on treatment approach (through traumatic laceration, bicoronal, or endoscopic transorbital). For the comparative analysis, cases with associated maxillofacial fractures or cerebrospinal fluid (CSF) leakage were excluded. Results: Out of 133 patients, 35 underwent surgery, with 6 patients treated using the endoscopic transorbital approach. This group of patients treated with the transorbital endoscopic approach demonstrated significantly shorter operative times compared to the bicoronal approach (mean 102 vs. 168 min, *p* = 0.021). They also had only minor complications, including temporary hypoesthesia and one transient ptosis. One patient had a minimal residual defect. The technique has been concluded to require endoscopic expertise. Conclusions: The endoscopic transorbital approach is a safe, minimally invasive alternative to the bicoronal approach for selected anterior wall frontal sinus fractures. Proper patient selection and surgical experience are essential to achieving favorable outcomes. Studies with longer follow-up are required to assess potential late complications, such as the development of mucoceles.

## 1. Introduction

Frontal sinus fractures represent a rare subset of facial fractures following maxillofacial trauma, with reported incidence rates in the literature ranging from 2% to 15% [1]. These fractures typically result from a high-velocity impact to the forehead and are often associated with other facial fractures [2]. They may involve only the anterior wall or both the anterior and posterior wall of the frontal sinus. An isolated posterior wall fracture is very uncommon [3,4]. The treatment of choice depends on the degree of displacement, the location, the involvement of other surrounding structures, the presence of cerebrospinal fluid (CSF) leakage, the involvement of the nasofrontal duct and the presence of secondary complications. As such, each case requires individualized assessment [2,5].

A general trend toward the conservative management of frontal sinus fractures has emerged in recent years. This approach is considered appropriate in cases where fracture fragments exhibit the following conditions: an absence of or only mild displacement, an absence of CSF leakage, and a nasofrontal duct that remains uninvolved [5]. When surgical intervention is indicated, the bicoronal approach remains the most widely recognized method for accessing the frontal sinus. However, this technique carries risks, including temporal hollowing, alopecia, injury to the frontal branch of the facial nerve and potential cosmetic deformities [6]. In response, the recent literature has proposed less invasive alternatives, such as the endoscopic transorbital approach [7].

The aim of this study was to evaluate the use of the minimally invasive transorbital endoscopic approach for reducing frontal sinus fractures and to compare this technique with the commonly used bicoronal approach. Additionally, we sought to establish procedural guidelines and provide clear indications for selecting this approach.

## 2. Materials and Methods

### 2.1. Study Design and Patient Selection

This retrospective single-center comparative analysis of case studies was conducted at the University Hospitals of Leuven, Belgium, and received ethical approval from the UZ/KU Leuven Ethics Committee on 20 February 2025 (reference number S70355). All patients presenting with a frontal sinus fracture diagnosed on CT scan between January 2013 and December 2023 were considered for inclusion. Patients were excluded if treatment had already been initiated at another hospital or if follow-up duration was less than one month.

Initially, patients were categorized into two groups: a conservative, non-surgical group and a surgical group. The surgical group was further subdivided into three categories based on the surgical approach employed: the bicoronal approach, the endoscopic transorbital approach, and approaches utilizing an existing traumatic laceration. The endoscopic transorbital approach was first used in our department in 2022. Since then, the majority of the isolated anterior wall fractures were treated with this technique.

For the comparative analysis between the bicoronal and endoscopic transorbital approaches, patients with CSF leakage or associated maxillofacial fractures were excluded as these conditions require more extensive surgical intervention, longer operative times and possibly a longer hospital stay, potentially introducing bias into the comparison. The diagnosis of CSF leakage was established based on CT scan findings, with an additional β2-transferrin test performed in cases of doubt.

### 2.2. Data Collection

The medical records of each included patient were reviewed to determine whether treatment would be conservative or surgical. Within the surgical group, additional data were extracted from the patient’s files. The following variables were collected: age, gender, fracture location (anterior and/or posterior wall), presence of additional maxillofacial fractures, presence of CSF leakage, involvement of the nasofrontal duct, duration of surgery, length of hospital stay, postoperative complications (including infection, bleeding, temporary or permanent hypoesthesia and temporary or permanent paralysis of the frontal branch of the facial nerve), visibility of scarring or residual defects and the need for additional surgical interventions.

### 2.3. Surgical Technique of the Endoscopic Transorbital Approach

Prior to initiating the surgical procedure, several anatomical landmarks are marked. It is advisable to outline the contours of the frontal defect before reduction, as identifying the original borders may become more difficult once manipulation has begun. In addition, the expected location of the supraorbital neurovascular bundle is marked, followed by the planned incision line. An upper blepharoplasty approach is commonly employed to access the anterior portion of the superomedial orbital wall.

**Figure 1:**
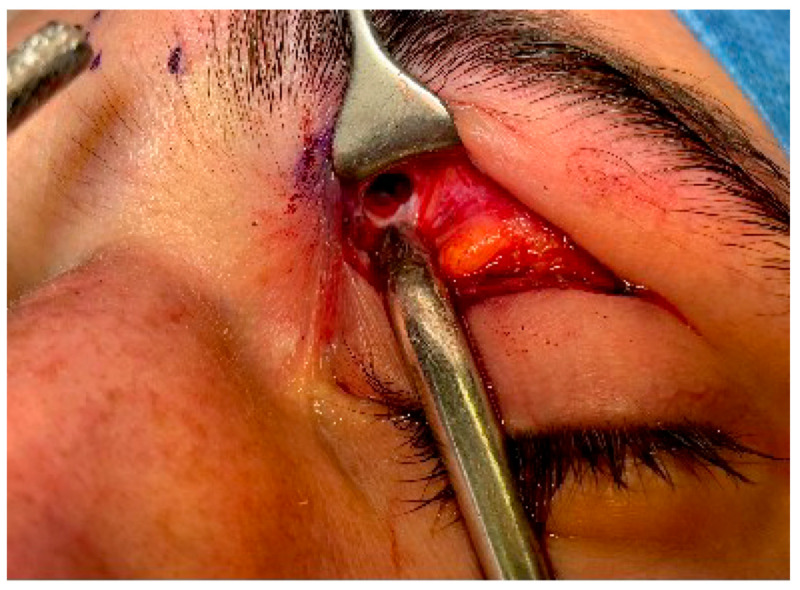
An upper blepharoplasty incision is used to reach the anterior part of the superomedial wall/corner of the orbit. At this point, a 5 mm bone window is created as an entrance for the endoscope and for a small instrument to perform the fracture reduction.

Upon bone exposure, a small periosteal incision is made to allow for an osteotomy using a round burr, creating a bone window of approximately 5 mm in diameter (Figure 1). This opening must be sufficiently wide to accommodate both the endoscope and a small instrument for fracture reduction (not simultaneously).

**Figure 2:**
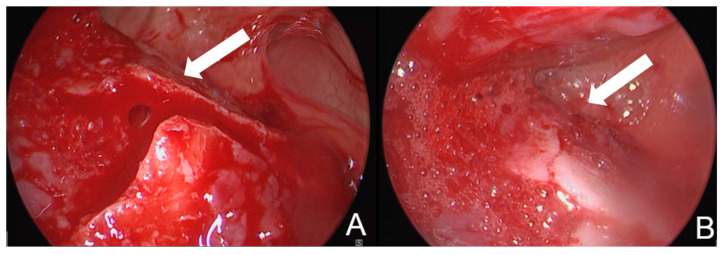
(**A**) Image of the fracture of the anterior wall of the frontal sinus before reduction; (**B**) image of the fracture of the anterior wall after reduction in the same patient. The anterior wall of the sinus is located at the bottom of the image. The fracture line is indicated with an arrow in both images.

A 2.7 mm 30° endoscope is inserted through the bony window to access the frontal sinus. This allows for direct visualization of the fracture prior to reduction. The fracture is then reduced using a small, rigid instrument such as an LPRF plugger or urethral dilator. Following reduction, the outcome is assessed both endoscopically and by manual palpation (Figure 2). If possible, a perioperative CBCT was taken to verify the reduction in the frontal sinus. In other cases, if no CBCT could be taken perioperatively, a control CT scan was made within 1 week after the procedure.

The wound is closed in layers, with particular attention given to the closure of the periosteum. The skin is closed using 6-0 Ethilon sutures, which are typically removed between five and seven days postoperatively.

### 2.4. Statistical Analysis

Statistical analysis was performed using the IBM SPSS Statistics Data Editor Version 29.0.2.0 (20). Descriptive statistics were performed for all included patients.

Comparative analyses between the selected surgical groups—excluding patients with associated maxillofacial fractures and/or CSF leakage—were performed using Fisher’s exact test and the Mann–Whitney U test. A significance level of 0.05 was applied.

## 3. Results

Over the 10-year inclusion period, a total of 133 patients presented with frontal sinus fractures. Of these, 98 patients were managed conservatively, while 35 underwent surgical treatment.

Within the surgical group, 32 patients were male and 3 were female. The mean age at the time of trauma was 38 years, with a range from 21 to 79 years. An isolated anterior wall fracture was observed in 19 patients, while 16 presented with combined anterior and posterior wall fractures. Surgical approaches included the bicoronal approach in 20 patients, access through an existing traumatic laceration in 9 patients and the endoscopic transorbital approach in 6 patients.

Following the exclusion of 16 patients with associated maxillofacial fractures and 3 patients with CSF leakage, the bicoronal group was reduced to 6 patients and the laceration group to 2 (Table 1).

After applying the exclusion criteria, all patients in the surgical group had isolated anterior wall fractures, except for one patient who presented with a combined anterior and posterior wall fracture. None of the included patients demonstrated involvement of the nasofrontal duct.

The mean duration of surgery was 168 min for the bicoronal approach and 102 min for the endoscopic transorbital approach. This difference was statistically significant (*p* = 0.021).

Regarding hospital stay, the majority of patients were discharged within two days. Only two patients had longer hospitalizations, lasting three and four days, both belonged to the bicoronal approach group. No statistically significant difference was observed in hospital stay between the groups.

In the bicoronal group, three patients reported temporary hypoesthesia of the supraorbital nerve at the first postoperative follow-up (one week). In one case, this evolved into hyperalgesia. No postoperative infections were noted; however, one patient required a secondary intervention due to postoperative bleeding.

**Figure 3:**
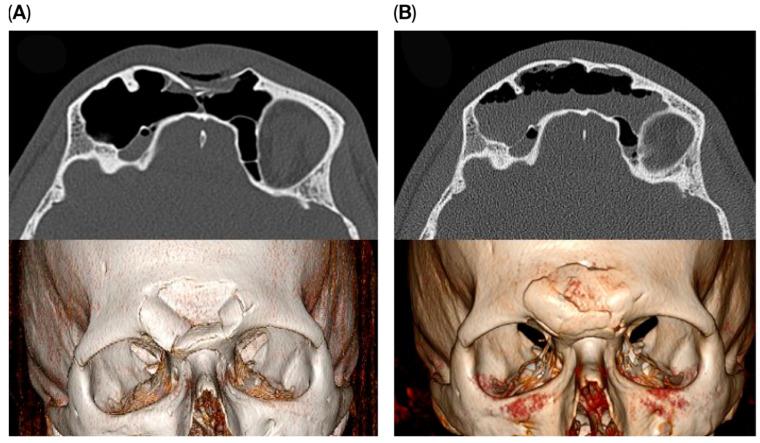
Pre- (**A**) and postoperative (**B**) images of a patient treated using the endoscopic transorbital approach.

Among patients treated via the endoscopic transorbital approach, four experienced temporary hypoesthesia of the supraorbital nerve one week postoperatively. All cases resolved within a few weeks. One patient developed temporary partial ptosis of the upper eyelid, which resolved approximately four weeks after surgery. In another patient, a residual defect of the anterior wall remained visible as a small external ‘dimple’. Although an additional corrective procedure was proposed, the patient opted against further intervention. All remaining patients achieved satisfactory fracture reduction with no visible residual defects (Figure 3).

**Figure 4:**
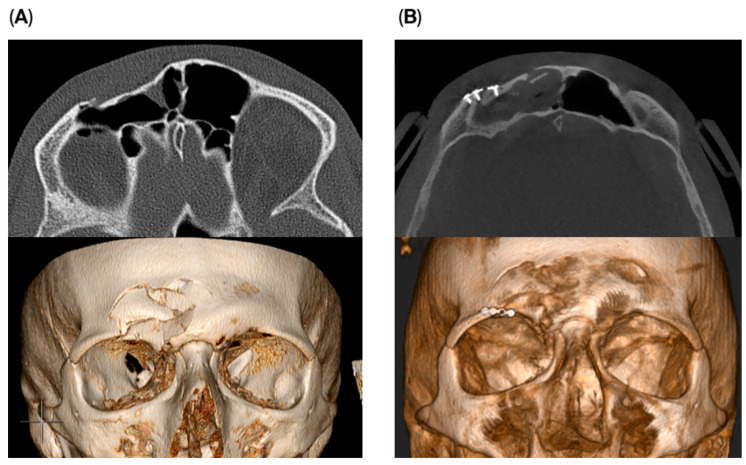
Pre- (**A**) and postoperative (**B**) images of a patient receiving the endoscopic transorbital approach with the use of osteosynthesis material for stabilization.

Osteosynthesis material was used in two cases within the endoscopic transorbital group to reconstruct the supraorbital rim and for stabilization of the fracture fragments. A small 1.5 plate was used to stabilize the fragments with fixation on the supra-orbital rim (Figure 4). The remaining four patients did not require osteosynthesis material. No impact on outcome was seen if osteosynthesis was used or not.

## 4. Discussion

At our institution, 26.3% of patients presenting with a frontal sinus fracture required surgical intervention. When a traumatic laceration was present in the surgical region, it was utilized as the access route for fracture treatment. In the absence of such a laceration, either a bicoronal or endoscopic transorbital approach was employed.

The endoscopic transorbital approach was performed in six cases. This technique demonstrated a significantly shorter operative time compared to the traditional bicoronal approach. It is important to note that these six cases represent the initial experience with this technique at our center. The learning curve associated with the approach was evident: the first procedure required 127 min, while the most recent was completed in just 72 min. These findings suggest that surgical time is likely to decrease further with increased experience. Despite the result being statistically significant, this should be interpreted with caution because of the small sample size of the study.

In addition to its time efficiency, the endoscopic approach involves a smaller incision. Although the incision is not hidden within the hairline, postoperative scarring was not visually prominent.

These above-mentioned advantages, together with the short hospitalization time after surgery, have a good influence on the cost-efficiency. The current literature supports an increasing preference for endoscopic techniques in the management of frontal sinus fractures, particularly when sinus preservation is feasible. In cases of extensive posterior wall involvement, especially when CSF leakage is suspected, sinus preservation is typically not possible. Under such circumstances, cranialization or obliteration can become necessary, and endoscopic approaches are not recommended [8].

In addition to the endoscopic transorbital approach, the endoscopic transnasal approach has also been described and may serve as an alternative, particularly in cases where complete avoidance of facial scarring is desired. However, a key limitation of the transnasal approach is the greater restriction of access to the frontal sinus, which makes a direct reduction in fracture fragments more challenging [9].

In cases involving obstruction of the nasofrontal duct, the transnasal approach may nonetheless offer added value and can be performed in combination with the transorbital approach. This dual approach provides an additional access portal, which may enhance both visualization and surgical manipulation [2].

The severity of complications associated with the endoscopic transorbital approach in our patient cohort was low. Four patients experienced temporary hypoesthesia of the supraorbital nerve, and one patient developed transient ptosis of the upper eyelid, which resolved within a few weeks. No major complications were observed during follow-up. It should be noted that because of the short follow-up period in our patient population, no assumptions can be made regarding late complications, such as mucoceles.

The current literature also supports the safety and growing adoption of endoscopic sinus-preservation techniques [10]. Some studies report even lower rates of postoperative complications, such as sinusitis or mucocele formation, in endoscopic surgery groups [8]. Theoretically, there remains a risk of unintentional intracranial penetration with surgical instruments, potentially causing CSF leakage [11]. A thorough preoperative assessment of fracture type, sinus anatomy and fracture extent is therefore critical to minimize this risk.

In our series, one out of six patients presented with a suboptimal reduction resulting in a visible residual defect. Similarly, Shumrick reported unsatisfactory outcomes in 7 out of 19 patients treated with a comparable endoscopic approach using a central incision and two lateral hairline incisions instead of an upper eyelid approach [12]. These potential esthetic limitations should be clearly discussed with patients during preoperative counseling and should be kept in mind when choosing for this approach. Moreover, the possibility of intraoperative conversion to an open bicoronal approach should be addressed if adequate reduction cannot be achieved endoscopically. This was always communicated to our patients in advance, but has not been necessary to perform to date. It is worth noting that open approaches typically require osteosynthesis with plates and screws, which can themselves alter forehead contour. Consequently, the clinical relevance of minor residual defects following the endoscopic transorbital approach remains open to debate.

Two patients required osteosynthesis material for fracture stabilization. In our opinion, this may be explained by the fact that in one patient, the fracture was more than one week old, while in the other case it may have been due to extensive stripping of the fracture fragments. However, given the very small sample size in our study, no definitive conclusions can be drawn.

In our experience, appropriate case selection is key to the success of this technique. The best outcomes were observed in patients with isolated anterior wall fractures presenting with a central dimple, where fragments could be repositioned effectively against the intact periosteal layer. In our department, this technique was not performed in cases with a simultaneous posterior wall fracture, to enhance the safety of the procedure. The literature however also supports the use of endoscopic techniques, where feasible, in managing posterior wall fractures—ideally in collaboration with neurosurgical colleagues [7].

The main limitation of this study is the small number of patients treated with the endoscopic transorbital approach. This reflects both the rarity of surgical indications for frontal sinus fractures and the frequent presence of concomitant facial fractures, which often necessitate a bicoronal approach. As a result, a degree of selection bias is unavoidable, given that the endoscopic transorbital technique is only suitable for a limited subset of fracture types.

Additionally, the retrospective nature of this study may have led to missing or incomplete data, particularly when details were not documented in the medical records. However, no missing data were observed in the variables analyzed. It should be noted, though, that four patients of the endoscopic transorbital approach group had only one month of follow-up. The other patients had a follow-up of two and four months. None of these patients presented to other departments or hospitals with complications related to their frontal sinus fracture treatment, according to our national patient registry.

Finally, further research is needed to validate these findings, ideally through larger prospective studies. With increasing experience in endoscopic surgery, it is expected that the indications for employing these minimally invasive techniques in frontal sinus fracture management will continue to expand. The transorbital approach offers fast and direct access to the frontal sinus, permits adequate instrument mobility for fracture reduction, and represents a promising alternative to both the bicoronal and endoscopic transnasal approaches.

## 5. Conclusions

In conclusion, the endoscopic transorbital approach for the treatment of frontal sinus fractures appears to be a safe, minimally invasive and time-efficient technique. In many cases, the use of osteosynthesis materials can be avoided. However, the procedure requires access to a 30° endoscope and familiarity with endoscopic instrumentation. Limitations include the potential for suboptimal fracture reduction due to restricted direct visualization and manipulation. Surgeons should be prepared for possible conversion to an open approach or consider secondary corrective procedures in the event of visible residual defects.

## Figures and Tables

**Table 1:** The included patients who received surgical treatment.

	Surgery Group	Surgery Group After Further Exclusion *
Bicoronal approach	20	6
Through existing laceration	9	2
Endoscopic transorbital approach	6	6

* This represents the number after the exclusion of patients with CSF leakage and/or other associated maxillofacial fractures.

## Data Availability

Data available on request due to privacy restrictions.
